# Micro RNA profiles in colostrum exosomes obtained from primiparous or multiparous dairy cows

**DOI:** 10.3389/fvets.2024.1463342

**Published:** 2024-10-30

**Authors:** Marta Terré, Anna Arís, Elena Garcia-Fruitós, Francesc Fàbregas, Alex Bach

**Affiliations:** ^1^Department of Ruminant Production, IRTA, Caldes de Montbui, Spain; ^2^Department of Animal and Veterinary Sciences, University of Lleida, Lleida, Spain; ^3^ICREA, Institut de Recerca i Estudis Avançats, Barcelona, Spain

**Keywords:** colostrum, dairy cow, exosome, miRNA, parity

## Abstract

Colostrum is rich in membranous vesicles of endocytic origin named exosomes, with proteins, lipids, RNA, and/or DNA cargos which can play different roles in physiological processes. Like other colostrum bioactive compounds, exosomes could be also influenced by individual characteristics. The objective of the study was to characterize miRNA cargo of colostrum exosomes from primiparous and multiparous cows in different farms. Twenty-seven colostrum samples of clinically healthy Holstein cows (11 primiparous and 16 multiparous) from 3 different farms were obtained and frozen. After thawing, exosomes were isolated following an ultracentrifugation protocol, and characterized morphologically. Particle size distribution and western immunoblotting were also analyzMaed. After RNA extraction, miRNAs were sequenced and analyzed to assess potential differences in profiles between primiparous and multiparous cows from different farms. Fourteen miRNA were upregulated and 11 miRNAs downregulated in primiparous compared with multiparous cows. Most of the miRNA differences between primiparous and multiparous cows regulate the gene expression of factors involved in mammary gland development and differentiation, and lipogenesis. In addition, miRNAs from one of the farms showed 8 miRNAs downregulated and 12 upregulated compared with the other 2 farms, independently of parity. Differences in miRNA between farms were mainly associated with immune and inflammatory-related genes. In conclusion, miRNA cargos of bovine colostrum exosomes differ in primiparous and multiparous cows, and some on-farm practices might also determine the content and activity of miRNA in colostrum exosomes.

## 1 Introduction

Colostrum is an important source of nutrients, and it is also a pivotal source of key hormones and growth factors that may act at both gastrointestinal and systemic levels (1). Colostrum quantity and quality depend on management and environmental factors such as season, calving ease, length of dry period, parity, temperature humidity index, and sex of calves (2), but also on nutritional (3) or metabolic health status of the dams (4). Colostrum quality research has mainly focused on immunoglobulins concentration and, to a lesser extent, on other molecules such as cytokines (5), hormones (3), nutrients (6) or immune cells (7). However, colostrum is also rich in membranous vesicles of endocytic origin, named exosomes, secreted by multiple cell types, found in all biofluids, and considered major players in cell–cell communication (8).

Exosomes can contain proteins, lipids, RNA, and DNA, participating in physiological and pathological processes. miRNAs are a group of small (~22 nucleotides) endogenous RNA molecules that regulate gene expression by binding coding RNA and blocking translation or methylation of promoter CpG-islands. There are more miRNAs in colostrum exosomes than in milk, and some of them are related to immune function and cell development miRNAs (9). It has been suggested that exosomes may participate in the development of the rumen (10), and region-specific miRNA have been found throughout the small intestine, whose expression patterns change throughout the first 6 weeks of the calf life (11). Interestingly, expression changes were most diverse during the first week of life, and many of these miRNAs targeted genes involved in the immune response, suggesting that the miRNA could regulate the development of the immune function during early life. In recent years, several studies have characterized bovine colostrum and milk microvesicles. Milk-isolated microvesicles carrying mRNAs and miRNAs have been related with calf gastrointestinal development and immune system (12). Furthermore, there exist differences in bovine colostrum miRNA between cow breeds (13), or between cows with different colostrum IgG concentration (14). We hypothesized that cow parity could influence colostrum miRNA in exosomes. Therefore, the objective of this study was to isolate colostrum exosomes and compare their miRNA cargos from primiparous and multiparous cows of different dairy farms.

## 2 Materials and methods

### 2.1 Sample collection

One hundred mL of unpasteurized bovine colostrum (first milking) was obtained after calving from 27 clinically healthy Holstein cows [11 primiparous and 16 multiparous (7 of second lactation, 5 of third lactation and 4 of forth lactation)] in 3 different farms all located in the north-east of Spain from July to November 2020. Samples were collected in sterile 50-mL conical tubes and immediately frozen at −20°C. All farms offered a single diet throughout the dry period, but its ingredient and nutrient composition differed by farm as described in Supplementary Table S1. All farms targeted 60-days for the length of the dry period, but actual duration differed among individuals within farms.

### 2.2 Exosomes isolation

Colostrum exosomes were isolated using ultracentrifugation based on (15) protocol with some modifications. After thawing the samples at room temperature, they were centrifuged twice at 12,500 × *g* for 15 min at 4°C and the upper fat layer removed. Then, samples were diluted 1:2 with PBS and centrifuged at 50,000 × *g* for 1 h at 4°C in a high-speed centrifuge. Then, four fifths of the supernatant were discarded (23 mL), and the remaining supernatant was washed in PBS for a final centrifugation at 50,000 × *g* for 2 h at 4°C in 38-mL ultracentrifuge tubes in a high-speed centrifuge. Then, the supernatant was discarded, and the smooth pellet formed on the top of the firm casein pellet was recovered and resuspended in PBS for exosome characterization analysis.

### 2.3 Exosomes characterization

#### 2.3.1 Cryo-transmission electron microscopy (Cryo-TEM)

Ultrastructural morphology of purified exosomes was analyzed by Cryo-Transmission Electron Microscopy (Cryo-TEM). A 3.9 μL drop of the purified exosomes samples were deposited onto holey carbon on a 400-mesh copper grid, previously treated by glow discharge. The grid was mounted on a plunger (Leica EM GP main unit, Leica Microsystems, Vienna, Austria), water excess was removed by blotting with filter paper (Whatman n°1 Filter Paper 90 mm Diameter, Silmid, Birmingham, UK) and exosomes suspension was straightforward vitrified by rapid immersion in liquid ethane (−178°C). Samples were mounted on a Gatan 626 cryo-transfer system and inserted into a Jeol JEM 2011 under cryo conditions. Electron microscope operating energy was 200 kV. Images were recorded using a Gatan Ultrascan US1000 CCD camera.

#### 2.3.2 Nanoparticle tracking analysis (NTA)

Particle size distribution and concentration were further assessed by Nanoparticle Tracking Analysis (NTA) with Nanosight NS300 (Malvern Panalytical, Malvern, UK). A 1:2,000–1:5,000 dilution of the sample in PBS was performed. Data were further processed using NanoSight Software NTA 3.4.

#### 2.3.3 Western immunoblotting

Purified exosomes samples were analyzed by western blot following the protocol previously described (16). Eight μL of purified exosomes were loaded per lane. Primary antibodies used were against CD9 (1:500, Invitrogen, Massachusetts), MFGE8 (1:1,000, Sigma, MO, USA), TSG101 (1:250, Invitrogen, MA, USA), and Alix (1:1,000, Cell Signaling Technology, MA, USA). Secondary anti-mouse Alkaline Phosphatase-conjugated antibody (1:20,000, Sigma, MO, USA) was used for CD9 and TSG101 and anti-rabbit Alkaline Phosphatase-conjugated antibody (1:30,000, Sigma, MO, USA) was used for MFGE8 and Alix. Positive control corresponding to bovine milk exosomes (Lyophilized Exosome Standards (Bovine Milk) ref: ESL-01, Creative Biolabs) was used.

#### 2.3.4 RNA sequencing and profiling

RNA from 200 μL of purified exosomes was extracted through the miRNeasy Serum/Plasma kit (Qiagen). RNA quality and quantity were assessed using an Agilent 2100 Bioanalyzer and RNA ladder. Small RNA libraries were prepared using the Small RNA_NebNext kit (NEB, Ipswich, MA, USA) according to the manufacturer’s instructions. Briefly, 1 μg of total RNA was subjected to DNase I treatment (RNase-Free) to remove genomic DNA contamination. RNA fragments of 10–40 nt in length were generated using the fragmentation buffer. The RNA fragments were then ligated with 3′ and 5′ adapters, followed by reverse transcription and PCR amplification to generate the sequencing library. The final libraries were analyzed using the Agilent 2100 Bioanalyzer. The libraries were sequenced on an Illumina HiSeq2500 with a single-end 50 bp read length according to the manufacturer’s instructions. The sequencing depth for each sample was 10 million reads.

### 2.4 Data analysis

The quality of the miRNA profiling raw data was checked using FastQC (17). The reads were trimmed for QC and the presence of the Universal Illumina adapter using Skewer v.2.0.0 (18). Trimmed reads were mapped to the *Bos taurus* reference genome (Ensembl, release 104, file Bos_taurus.ARS-UCD1.2.dna.fa.gz) using Bowtie v.1.2.2 (19) and counts were assigned to annotated miRNA genes (Ensembl, release 104, file Bos_taurus.ARS-UCD1.2.104.chr.gtf.gz) using HTSeq v.2.0 (20). The number of unaligned tags was increased to the refinement made using ShortStack v.3 (21) by removing the multi-mapping reads impossible to reassign. Qualimap v.2.2.1 (22) was used to check the quality of the aligned reads. Differential expression analysis was performed in R (version 4.0.0) (23)/Bioconductor (24) environment, using the DESeq2 package version 1.28.1 (25). Genes that had less than 10 read counts across all samples were filtered out before processing the data. Principal component analysis (PCA) was conducted using the prcomp method from the stats core R package on the regularized log-transformed data and plotted using the ggplot2 package (26). First parity or other parities were the fixed effect, and farms were considered independent among them. Samples 604, 6,598, and 9,280 were removed from the analysis as outliers. The expression of miRNAs genes between type of animals (primiparous vs. multiparous) within farms were selected as differentially expressed if the FDR-adjusted *p*-value was less than 0.05, and the absolute log2 fold change was more than 1. In addition, bedtools v2.27.1 (27) and samtools 1.8 (28) were used. Similarly, the different expressions of miRNAs among the three farms, compared by pairs, were evaluated following the same procedure.

## 3 Results

### 3.1 Exosome characterization

**Figure 1:**
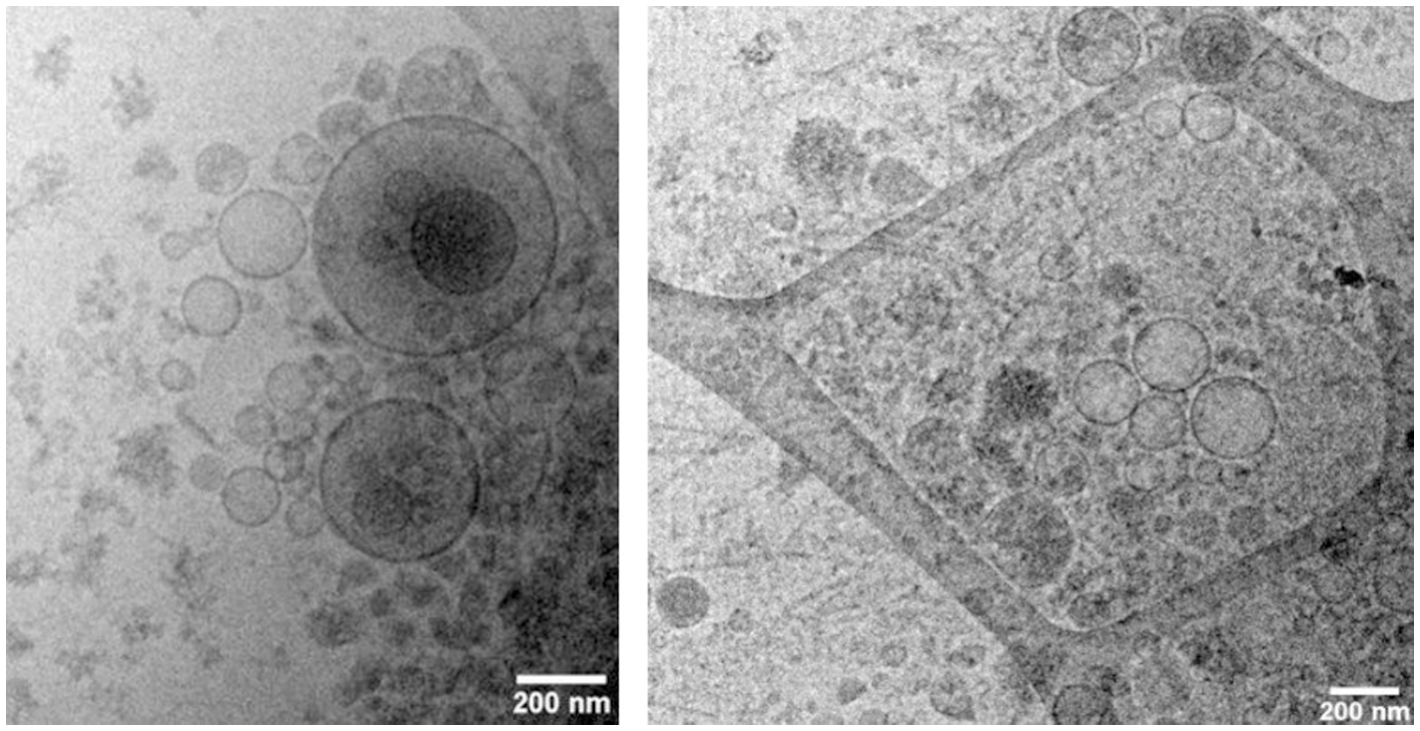
Bovine colostrum exosomes visualized by CryoTEM.

**Figure 2:**
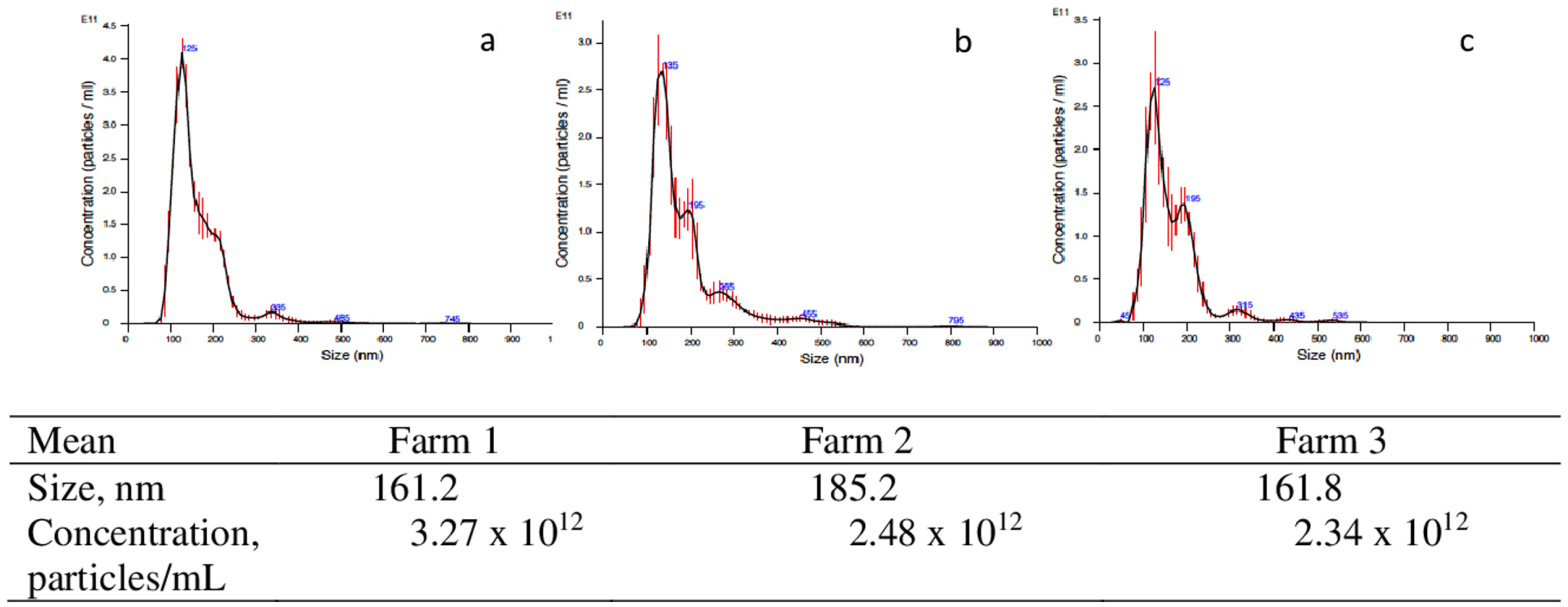
Examples of nanoparticles size distribution of isolated exomes samples from primiparous cows in Farm I (a), Farm 2 (b), and Farm 3 (c).

**Figure 3:**
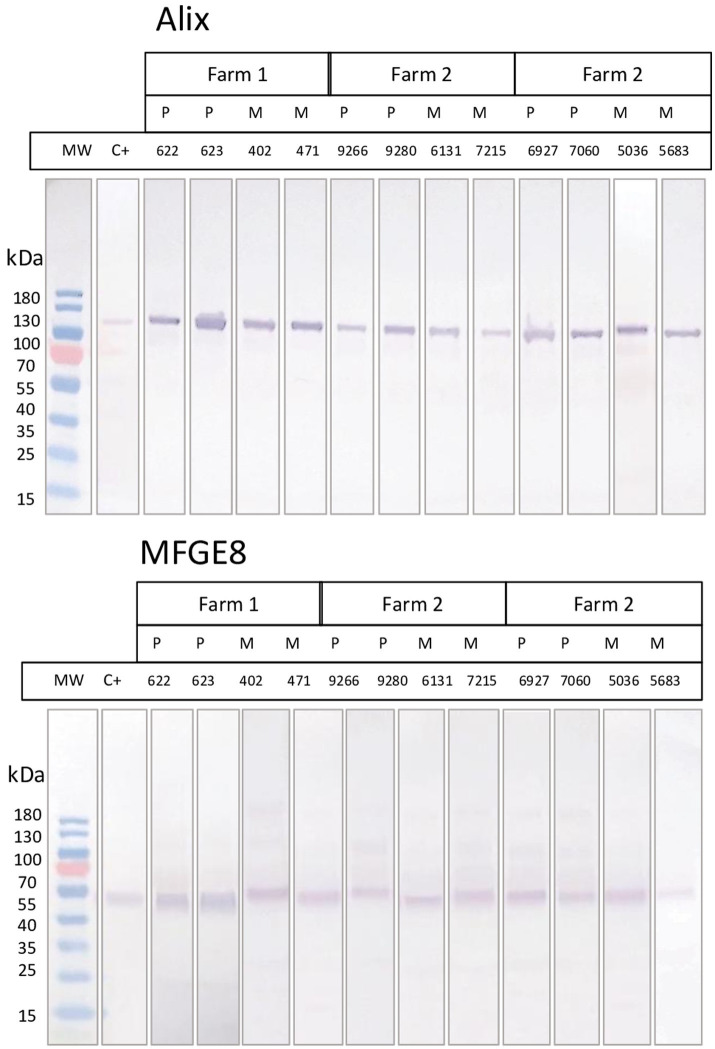
Western blot analyses of the presence of two exosome markers (Alix and MFGE8) in samples of either primiparous or multiparous cows of the 3 farms included in the study. Positive control corresponds to commercial purified exosomes from bovine milk (Lyophilized Exosome Standards, Creative Biolabs).

Bovine exosomes were characterized by Cryo-TEM, NTA, and Western immunoblotting. Images of Cryo-TEM showed that colostrum vesicles had a round appearance (Figure 1). No differences were observed in particle size or yield either in primiparous or multiparous cows, or among the different farms (Figure 2). The mean particle size of all samples was 182 ± 8.3 nm with a main peak at 125–135 nm, and they were within the 10 and 90 percentile range of 111 ± 2.4 nm and 276 ± 10.6 nm, respectively, and mean particle yield was of 1.97 ± 0.230×10^12^ particles/mL. Western blot confirmed that samples were enriched with exosome-specific markers MFGE8 and ALIX (Figure 3). Marker CD9 was not detected in colostrum samples whereas TSG101 was detected in all analyzed samples but not in the positive control used (Supplementary Figure S1).

### 3.2 Characterization of miRNAs

A total of 943 miRNAs were isolated, with 88.4% of them associated with known genes. Twenty miRNAs represented between 80 and 85% of total miRNAs reads, from those, 12 were known (*bta-miR-30a, bta-miR-26a-2, bta-miR-99a, bta-let-7f-2, bta-miR-200a, bta-let-7a-3, bta-miR-2285 t, bta-miR-200c, bta-miR-27b, bta-miR-30d, bta-miR-30f, bta-let-7a-2*). The comparison between primiparous and multiparous cows revealed 14 miRNAs downregulated (*bta-miR-2478, bta-miR-885, bta-miR-503, bta-miR-708, bta-miR-424, bta-miR-9-1, bta-miR-218-1, bta-miR-452, bta-miR-497, bta-miR-195, bta-miR-3431, bta-miR-378c*, *bta-miR-224,* and *bta-miR-181c*, in order of log2Folds magnitude), and 11 miRNAs upregulated (*bta-miR-128-2, bta-miR-143, bta-miR-145, bta-miR-181a-1, bta-miR-181b-2, bta-miR-1388, bta-miR-181a-2, bta-miR-193a, bta-miR-2299, bta-miR-28*, and *bta-miR-10174*, in the order of 2 log-folds) in primiparous in comparison with multiparous cows. The overview of these differences was represented in a heatmap as Supplementary Figure S2.

**Figure 4:**
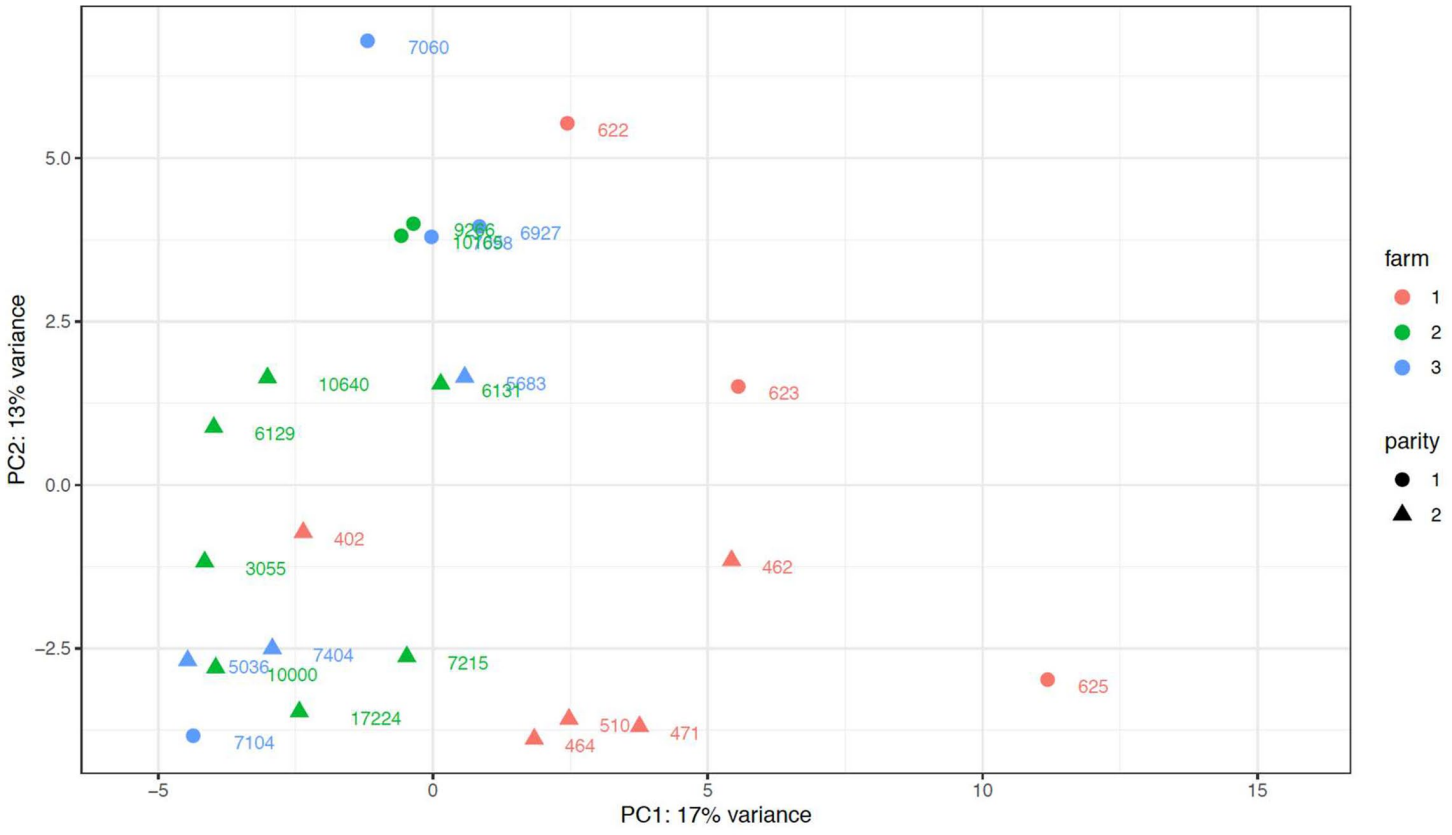
Principal Component Analysis plot of miRNA of exomes isolated from colostrum bovine samples from primiparous (parity l) and multiparous (parity 2) cows from three different dairy farms. Parity 2 includes parities from 2 to 4 lactations (animal ID of 2 lactations: 510, 6,129, 6,131, 6,598, 7,404, 10,000, 10,640, animal ID of 3 lactations: 462, 464, 471, 5,683, 7,215, animal ID of4 lactations: 402, 3,055, 5,036, 17,224).

When assessing potential anecdotical differences in colostrum samples among farms, one farm was distinguished in the PCA plot from the other 2 farms (Figure 4), and it was also revealed in the DESeq2 analysis. Colostrum samples from the outstanding farm had 8 downregulated (*bta-miR-99a, bta-miR-181d, bta-miR-126, bta-miR-195, bta-miR-2387, bta-miR-2285e-1,* and 2 unknown miRNAs in chromosome 7and 8), and 12 upregulated (*bta-miR-221, bta-miR-146a, bta-miR-345, bta-miR-222, bta-miR-142, bta-miR-155, bta-miR-223, bta-miR-2284w, bta-miR-378c, bta-miR-378-2* and 2 unknown miRNAs in chromosome 7 and 19) miRNAs compared with the other 2 farms, which did not differ in the expression of any miRNAs.

## 4 Discussion

Herein mean particle size of exosomes (182 ± 8.3 nm) was greater than that described in the literature in colostrum samples (149 ± 8.6 nm) (29) or in milk samples (100 nm) (30). The mean particle yield was of 1.97 ± 0.230×10^12^ particles/mL, which was also greater than that reported in the literature (1.4×10^11^ particles/mL) (31), probably mainly due to different exosome isolation protocols. In the current study, several filtration procedures (filtration using cellulose acetate filters (1′2, 0′8 and 0′45 mm) or PVDF filters (0′45 mm), dialysis membranes with a 300 kDa cut-off, and centrifuge filters with a 300 kDa cut-off) were tested without affecting the quality of exosomes (Supplementary Figure S3). By contrast, previous studies reported a benefit of using filter membranes before (29, 31) or after (30) ultracentrifugation, or using a size exclusion chromatography column after centrifugation (31). The lack of filtration steps in the current samples may have increased particle size and number of exosomes in comparison with previous studies. Similar to our study, TSG101 and Alix were found in colostrum samples (8), and MFGE8 was detected in bovine raw milk (32). Colostrum samples herein did not mark for CD9, which it has been previously reported to be present in exosomes from bovine milk (29, 31), and bovine transition milk samples collected during the first and second days in milk of lactating cows (33).

The highly expressed miRNAs found were common among all animals and farms, and they have also been reported as common miRNA in milk or colostrum samples in previous studies (34, 35). Some of the different miRNA contents in primiparous and multiparous cows regulate the expression of genes participating in mammary gland development and differentiation. The *bta-miR-424/503* cluster and *bta-miR-452* were downregulated in primiparous cows, and they have been described to be up-regulated during mammary gland involution (36, 37). Specifically, *bta-miR-424/503* cluster has been reported to be involved in the transcriptional regulation of TGF-ß (36). Furthermore, *bta-miR-708* was also downregulated in primiparous cows, and it has been reported to regulate the expression of TNFSF11, EGF, and HOXA5, which are genes involved in the development and differentiation of the mammary gland (38). Lastly, *bta-miR-128-2* was upregulated in primiparous cows, and it has been described to be repressed by TGF-ß during mammary epithelial oncogenic transformation (39). Some other miRNAs that differed between primiparous and multiparous cows have been involved in lipogenesis. On one hand, some were downregulated in primiparous cows: *bta-miR-497*, which has been involved in the regulation the fatty acid synthesis (40), *bta-miR-378c*, which is related to maternal body conditional index (41), and *bta-miR-*3431 which has been reported to be upregulated in dairy cows when they were supplemented with linseed oil (42). On the other, the miRNA upregulated in primiparous cows were *bta-miR-193a,* which has been involved in adipocyte differentiation (43), *bta-miR-181a-1, bta-miR-181b-2, bta-miR-143,* and *bta-miR-145*, which have been involved in milk fat synthesis (44–47), and *bta-miR-2299* and *bta-miR-1388,* which they were downregulated and *bta-miR-28,* which was upregulated, in dairy cows when they were supplemented with linseed or safflower oils (42). Other miRNAs downregulated in primiparous cows were *bta-miR-2478,* which has been associated with the inhibition of melanin production (48), *bta-miR-885* which has been related with retinol biosynthesis (37), and two miRNAs (*bta-miR-195,* and *bta-miR*-*181c*) linked to tumor growth signaling pathways (49, 50).

Although it was not the objective of the study and animals were only checked by clinical diseases signs and clinical mastitis, we observed that miRNA profile of colostrum exosomes of one farm differed from the other 2 in the PCA. The miRNA found to differ upregulate genes related to immune and inflammatory responses like in *Staphylococcus aureus* infections [*bta-miR-378-2* and *bta-miR-223*; (51, 52), respectively], in the regulation of lymphocyte functions [*bta-miR-155*; (53)], or as biomarkers in inflammatory diseases [*bta-miR-221* and *bta-miR-222*; (54)]. Those miRNAs that were downregulated in that specific farm were more diverse in their regulation responses: *bta-miR-126* was found to regulate vascular integrity (55), *bta-miR-181d* has been involved in the regulation of inflammatory response in heat-stressed cows (56), or *bta-miR-2387* targeted lipid metabolism-related genes in (57) study. Although the study of differences among farms was exploratory, and it was not targeting any specific farm management practice or animal diseases, the fact that 2 farms had similar miRNA profiles (while all farms differed in nutrients and ingredients composition, Supplementary Table S1), may exclude diet composition as an influencing factor on colostrum exosomes miRNAs in the present study. Since most of the upregulated mi-RNAs in the outstanding farm were immune and inflammatory related, and this farm was not vaccinating cows during the dry period while the other 2 farms did, vaccination might be an important factor behind this anectodical difference in miRNA composition of colostrum.

Exosomes in colostrum from primiparous and multiparous cows differed in 25 miRNAs. Most of these miRNAs are involved in the regulation of lipogenic pathways, and development and differentiation of the mammary gland.

## Data Availability

Sequencing data are available in the NCBI BioProject under accession number PRJNA1165141.
