# Supplementary material for: Micro RNA profiles in colostrum exosomes obtained from primiparous or multiparous dairy cows
Source: Front Vet Sci. 2024 Oct 30;11:1463342. doi: 10.3389/fvets.2024.1463342 (PMC11561390; doi:10.3389/fvets.2024.1463342)
Supplement: Supplementary file 1 [file Data_Sheet_1.PDF]

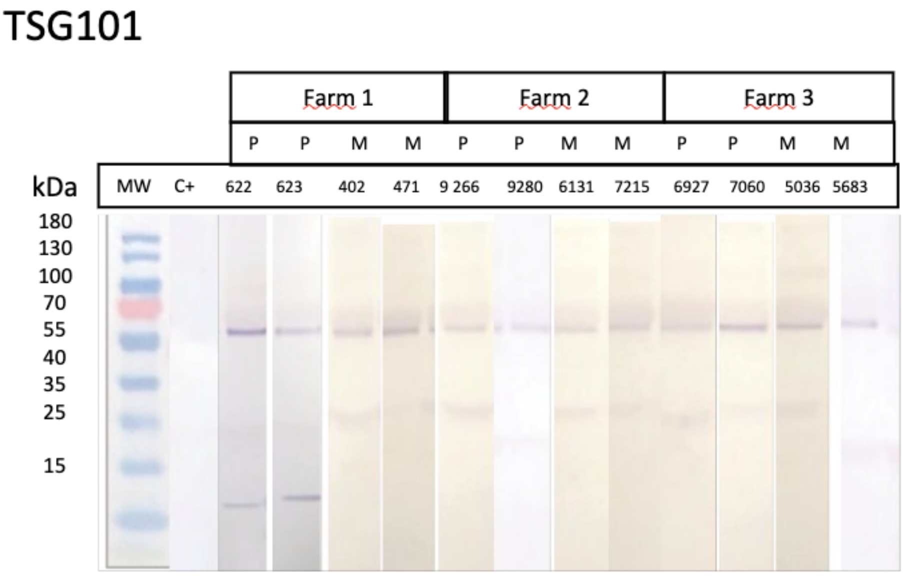

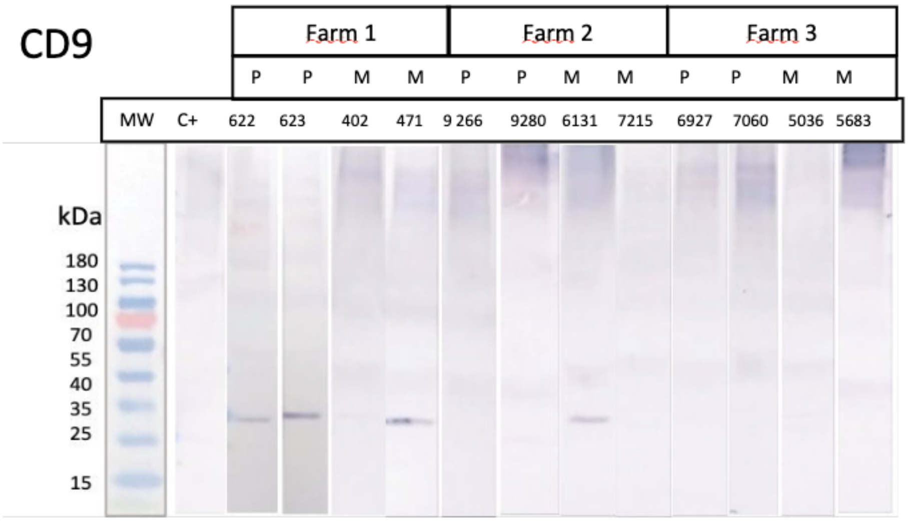

Supplementary material. Western blot analyses of the presence of two exosome markers (TSG101 and CD9) in samples of either primiparous or multiparous cows of the 3 farms included in the study. Positive control corresponds to commercial purified exosomes from bovine milk (Lyophilized Exosome Standards, Creative Biolabs).
