# Supplementary material for: Micro RNA profiles in colostrum exosomes obtained from primiparous or multiparous dairy cows
Source: Front Vet Sci. 2024 Oct 30;11:1463342. doi: 10.3389/fvets.2024.1463342 (PMC11561390; doi:10.3389/fvets.2024.1463342)
Supplement: Supplementary file 2 [file Data_Sheet_2.PDF]

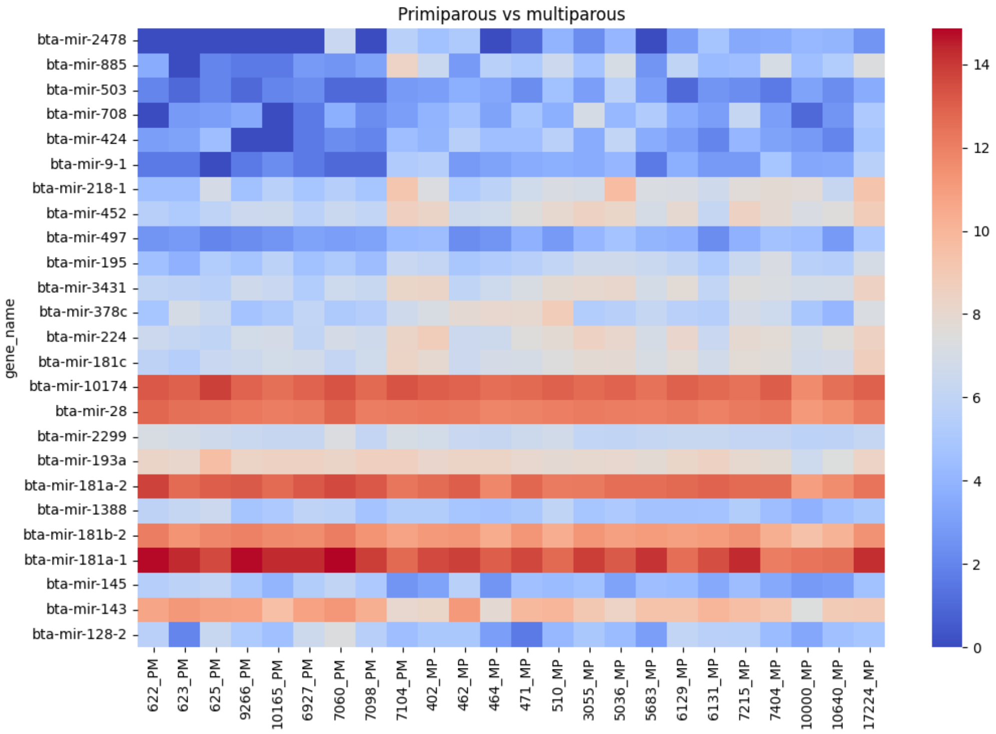

Supplementary Figure 2. Heatmap of significant genes of bovine colostrum from primiparous (PM) and multiparous (MP) dairy cows.
