# Supplementary material for: Micro RNA profiles in colostrum exosomes obtained from primiparous or multiparous dairy cows
Source: Front Vet Sci. 2024 Oct 30;11:1463342. doi: 10.3389/fvets.2024.1463342 (PMC11561390; doi:10.3389/fvets.2024.1463342)
Supplement: Supplementary file 3 [file Data_Sheet_3.PDF]

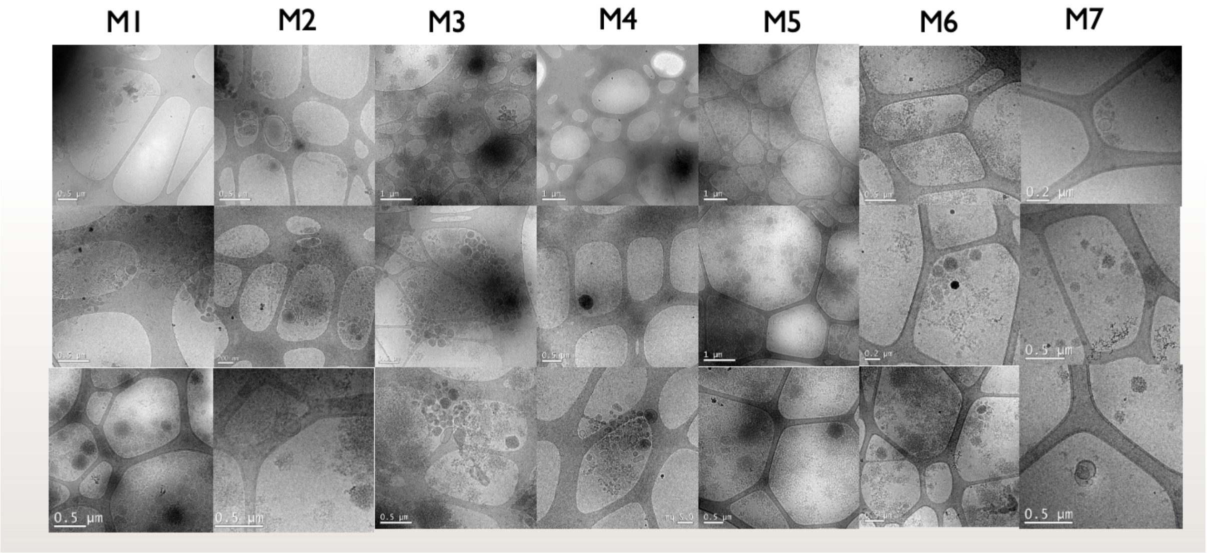

Supplementary Figure 3. CryoTEM images of exosomes using 6 different methods: M1) Exoquick™ method, M2) Ultracentrifugation method, M3) Exoquick™ followed by the ultracentrifugation method, M4) Ultracentrifugation followed by Exoquick™ method, M5) rennet pre-cleaning protocol followed by Exoquick™ method, M6) rennet pre-cleaning protocol followed by ultracentrifugation method, M7) Diafiltration
