# Supplementary material for: Micro RNA profiles in colostrum exosomes obtained from primiparous or multiparous dairy cows
Source: Front Vet Sci. 2024 Oct 30;11:1463342. doi: 10.3389/fvets.2024.1463342 (PMC11561390; doi:10.3389/fvets.2024.1463342)
Supplement: Supplementary file 4 [file Data_Sheet_4.PDF]

Supplementary Table 1. Nutrient composition of the prepartum rations and milk tank quality of farms enrolled in the study.

Item	Farm 1	Farm 2	Farm 3
Ingredient composition, %			
Alfalfa silage	2.14	-	-
Wheat silage	1.21	-	-
Rye-grass silage	-	1.84	-
Rye-grass hay	-	6.16	-
Fescue hay	-	-	3.36
Straw	6.49	-	5.21
Corn meal	1.29	1.06	1.26
Wheat meal	1.90	-	0.46
Barley meal	-	-	0.46
Soybean hulls	-	-	0.46
Soybean meal	-	0.79	0.41
Canola meal	1.40	-	-
Magnesium oxide	0.03	-	-
Premix[1]	0.04	0.30	0.9
Nutrient, %			
DM	67.0	55.1	57.9
CP	11.8	13.7	10.4
EE[1]	2.3	3.2	1.6
NDF	49.7	50.1	57.4
ADF	33.2	32.4	36.3
Ash	7.3	10.3	6.7
Calcium	0.44	1.08	0.80

[1] Vitamin and mineral mix differed among farms. Farm1 composition: VitaminD_3 800,000 UI/kg, Vitamin A 2,240,000 IU/kg, Vitamin E 8,000 mg/kg, citric acid 5 mg/kg, Zn 26,000 mg/kg, Co 40 mg/kg, Cu 5,000 mg/kg, I 240 mg/kg, Fe 20,000 mg/kg, Mn 24,000 mg/kg, Se 160 mg/kg; Farm2 used an anionic salt premix; Farm3 composition: 50% corn, 21% barley, 10% calcium carbonate, 8% wheat, 4% salt, 3.5% magnesium oxide, 2% vitamins and minerals premix from lactating cows, 1% palm oil, 0.32% cobalt, 0.08% zinc
